# All-Trans Retinoic Acid Induces Differentiation and Downregulates Stemness Markers and MGMT Expression in Glioblastoma Stem Cells

**DOI:** 10.3390/cells14100746

**Published:** 2025-05-20

**Authors:** Justin Tang, Raymond Yang

**Affiliations:** 1 Department of Biomedical Science, University of Guelph, Guelph, ON N1G 2W1, Canada; 2 Department of Surveillance & Evaluation, Health Canada, Ottawa, ON K1A 0K9, Canada

**Keywords:** glioblastoma, cancer stem cells, established cell lines, U87-MG, A172, all-trans retinoic acid (ATRA), differentiation, stemness, SOX2, nestin, MGMT, temozolomide resistance, gene expression, quantitative real-time PCR (qPCR)

## Abstract

Background: Glioblastoma (GBM) remains almost uniformly fatal, owing in part to therapy-resistant cancer stem-like cells (CSCs) and to temozolomide (TMZ) resistance driven by O^6^-methylguanine-DNA methyltransferase (MGMT). Differentiation therapy with all-trans retinoic acid (ATRA) has the potential to attenuate stemness and sensitize GBM to TMZ. We therefore asked whether ATRA reduces expression of key CSC markers and MGMT in established GBM lines. Methods: Two established human GBM cell lines, U87-MG and A172, were cultured under neurosphere-promoting conditions to enrich for potential stem-like subpopulations. Cells were treated with either 1 µM ATRA or vehicle control (DMSO) for 5 days. Total RNA was extracted, and cDNA was synthesized. Quantitative Real-Time PCR (qPCR) assessed relative mRNA expression levels of key stemness transcription factors (SOX2, NES) and the DNA repair gene MGMT and corresponding protein levels were measured by an Enzyme-Linked Immunosorbent Assay (ELISA). Gene expression was normalized to the geometric mean of two validated housekeeping genes (GAPDH, ACTB). Relative quantification was calculated using the ΔΔCt method, and statistical significance was determined using Student’s *t*-tests. Results: ATRA markedly suppressed stemness and MGMT in both lines. In U87-MG, SOX2 mRNA fell 3.7-fold (*p* = 0.0008) and protein 2.99-fold (148.3 ± 6.0 → 49.7 ± 2.7 pg µg^−1^; *p* = 0.0002); Nestin dropped 4.1-fold (*p* = 0.0005) and 3.51-fold (450.0 ± 17.3 → 128.3 ± 4.4 pg µg^−1^; *p* = 0.00008). MGMT decreased 2.6-fold at transcript level (*p* = 0.0065) and 2.11-fold at protein level (81.7 ± 4.4 → 38.7 ± 1.8 pg µg^−1^; *p* = 0.0005). In A172, SOX2 was reduced 2.9-fold (*p* = 0.0041) and 2.31-fold (*p* = 0.0007); Nestin 3.3-fold (*p* = 0.0028) and 2.79-fold (*p* = 0.00009). MGMT declined 2.2-fold (*p* = 0.0132) and 1.82-fold (*p* = 0.0015), respectively. Conclusions: Five-day exposure to ATRA diminishes SOX2, Nestin, and MGMT at both mRNA and protein levels in stem-enriched GBM cultures, supporting the premise that ATRA-induced differentiation can concurrently blunt CSC traits and TMZ-resistance mechanisms. These data provide a molecular rationale for testing ATRA in combination regimens aimed at improving GBM therapy.

## 1. Introduction

Glioblastoma (GBM) is the most common and aggressive primary malignant brain tumor in adults, associated with a dismal prognosis despite multimodal therapy comprising surgery, radiation, and chemotherapy [1,2]. The inherent challenges in treating GBM stem from its aggressive invasiveness, profound intra- and inter-tumoral heterogeneity, and remarkable capacity for therapy resistance leading to near-universal recurrence [3,4]. A subpopulation of cells within the tumor, often exhibiting stem-like properties (cancer stem-like cells or CSCs), is increasingly recognized as a central driver of these malignant characteristics. These cells possess self-renewal capabilities, potential for multi-lineage differentiation, and are preferentially resistant to conventional therapies, enabling them to initiate tumor formation and repopulate the tumor mass after treatment [5,6,7]. Key transcription factors and cytoskeletal proteins, such as SOX2 (SRY-Box Transcription Factor 2) and Nestin (NES), are associated with this stem-like state and are often used as markers [8,9]. Targeting pathways active in these subpopulations represents a critical unmet need for improving GBM patient outcomes.

Temozolomide (TMZ), an alkylating agent, is the cornerstone of current GBM chemotherapy [10]. TMZ exerts its cytotoxic effect primarily by methylating DNA bases, particularly at the O^6^ position of guanine (O^6^-MeG). This lesion, if unrepaired, leads to DNA double-strand breaks during replication and triggers cell cycle arrest and apoptosis [11]. However, the efficacy of TMZ is frequently limited by intrinsic and acquired resistance mechanisms. The most significant factor mediating TMZ resistance is the DNA repair enzyme O^6^-methylguanine-DNA methyltransferase (MGMT) [12,13]. MGMT directly removes the methyl group from O^6^-MeG, thereby reversing the cytotoxic lesion and conferring resistance. High MGMT expression levels in tumor cells strongly correlate with poor response to TMZ and reduced patient survival [14]. Epigenetic silencing of the MGMT promoter via methylation is a predictive biomarker for TMZ sensitivity, but many GBMs exhibit high MGMT expression due to an unmethylated promoter, posing a major clinical challenge [15,16]. Strategies to overcome MGMT-mediated resistance are urgently needed.

Differentiation therapy, aiming to shift cancer cells from a resistant stem-like state to a more mature phenotype, offers a potential therapeutic strategy [17]. All-trans retinoic acid (ATRA), a vitamin A metabolite and established differentiation agent [18], functions via nuclear retinoic acid receptors (RARs) to regulate genes involved in differentiation and growth [19]. In glioma models, ATRA has shown potential to induce differentiation and inhibit proliferation [2,20,21]. A critical question is whether ATRA-induced differentiation can also modulate key chemoresistance mechanisms, such as MGMT expression, particularly in GBM cells exhibiting stem-like features. While some studies suggest ATRA might sensitize glioma cells to chemotherapy [22,23], the direct impact of ATRA on MGMT expression in conjunction with stemness marker reduction in GBM models enriched for stem-like properties remains a key area for investigation.

Therefore, we hypothesized that ATRA treatment would not only reduce the expression of stemness markers (SOX2, NES) but also concurrently decrease the transcript and protein levels of the chemoresistance gene MGMT in established human GBM cell lines cultured under neurosphere conditions. To test this, we utilized U87-MG and A172 cell lines, assessing mRNA expression of SOX2, NES, and MGMT via qPCR and their corresponding protein levels via ELISA following ATRA treatment. Our findings aim to provide molecular evidence linking ATRA-induced differentiation to the modulation of both stemness and MGMT expression in these GBM models.

## 2. Materials and Methods

### 2.1. Cell Lines and Culture Conditions

Established human glioblastoma cell lines U87-MG (ATCC^®^ HTB-14™) and A172 (ATCC^®^ CRL-1620™) were obtained from the American Type Culture Collection. To enrich for potential stem-like subpopulations and facilitate comparison with stem cell studies, cells were cultured as non-adherent neurospheres in serum-free NeuroCult™ NS-A Proliferation Medium supplemented with NeuroCult™ Proliferation Supplement (STEMCELL Technologies, Vancouver, BC, Canada), 20 ng/mL recombinant human epidermal growth factor (EGF; PeproTech, Dollard-des-Ormeaux, QC, Canada), 10 ng/mL recombinant human basic fibroblast growth factor (bFGF; PeproTech), and 1% penicillin-streptomycin (Thermo Fisher Scientific, Mississauga, ON, Canada). Cells were maintained in uncoated T75 flasks (Corning Life Sciences, Ottawa, ON, Canada) at 37 °C in a humidified atmosphere containing 5% CO_2_. Neurospheres were passaged every 5–7 days by mechanical dissociation followed by enzymatic dissociation using Accutase (STEMCELL Technologies, Cedarlane Labs, Vancouver, BC, Canada) for 5–7 min at 37 °C, followed by quenching with medium, centrifugation (300× *g*, 5 min), and resuspension in fresh medium. Cell lines were routinely tested for mycoplasma contamination using a PCR-based detection kit (MycoAlert™, Burlington, ON, Canada).

### 2.2. All-Trans Retinoic Acid (ATRA) Treatment

All-trans retinoic acid (Sigma-Aldrich, Oakville, ON, Canada) was prepared as a 10 mm DMSO stock and stored light-protected at −80 °C. For experiments, cells were dissociated into single cells as described above and seeded at a density of 1 × 10^5^ cells/mL in 6-well plates (Corning) containing 2 mL of complete NeuroCult medium per well. Cells were allowed to recover for 24 h before treatment. ATRA stock solution was diluted in culture medium to achieve a final concentration of 1 µM. Vehicle control wells received an equivalent volume of DMSO (final concentration 0.01%). Treatments were performed in biological triplicate for each cell line and condition. Cells were incubated with ATRA or vehicle for 5 days at 37 °C and 5% CO_2_, with fresh medium containing the respective treatment added after 2.5 days. All procedures involving ATRA were performed under subdued light conditions. The concentration (1 µM) and duration (5 days) were selected based on established literature demonstrating ATRA-induced effects in glioma cells and were confirmed in preliminary viability assays (e.g., using Trypan Blue exclusion or a standard MTS/WST assay) to exhibit minimal cytotoxicity under these specific neurosphere culture conditions, suggesting observed gene and protein expression changes are primarily due to differentiation rather than overt toxicity. While these preliminary checks are against broad cytotoxicity, future studies could incorporate specific apoptosis markers (e.g., cleaved caspase-3, Annexin V staining) to further delineate differentiation effects from potential low-level cytotoxic contributions to the observed gene and protein expression changes.

### 2.3. RNA Isolation and Quality Control

Total RNA was isolated from collected neurospheres using the RNeasy Mini Kit (Qiagen, Toronto, ON, Canada) with on-column DNase I digestion, per the manufacturer’s instructions. RNA concentration and purity (A260/280 and A260/230 ratios) were assessed using a NanoDrop™ 2000 spectrophotometer (Thermo Fisher Scientific), and integrity was confirmed for representative samples by agarose gel electrophoresis.

### 2.4. cDNA Synthesis

First-strand complementary DNA (cDNA) was synthesized from 1 µg of total RNA using the High-Capacity cDNA Reverse Transcription Kit (Applied Biosystems, Thermo Fisher Scientific) in a 20 µL reaction volume, according to the manufacturer’s instructions. The reaction mixture included random primers and MultiScribe™ Reverse Transcriptase. Reactions were performed in a thermal cycler (Veriti™ 96-Well Thermal Cycler, Applied Biosystems, Thermo Fisher Scientific) with the following program: 25 °C for 10 min, 37 °C for 120 min, and 85 °C for 5 min. Control reactions lacking reverse transcriptase (-RT controls) were prepared for representative samples to verify the absence of significant genomic DNA amplification during subsequent qPCR. Synthesized cDNA was diluted 1:5 with nuclease-free water (Thermo Fisher Scientific) and stored at −20 °C until use.

### 2.5. Quantitative Real-Time PCR (qPCR)

Primers for human SOX2, NES, MGMT, GAPDH, and ACTB were designed using Primer3Plus software (version: 3.3.0) and their sequences are listed in Table 1 (Integrated DNA Technologies). All primer pairs were validated for amplification specificity (single peak in melt curve analysis following each qPCR run) and efficiency (confirmed to be between 90 and 110% using standard curves from serial dilutions of pooled cDNA).

Each 10 µL qPCR reaction contained 5 µL of PowerUp™ SYBR™ Green Master Mix (2×), 0.5 µL of forward primer (10 µM stock), 0.5 µL of reverse primer (10 µM stock), 2 µL of diluted cDNA (corresponding to 20 ng of initial RNA input), and 2 µL of nuclease-free water. Reactions were performed in technical triplicate for each biological replicate. Standard thermal cycling conditions were used: UDG activation at 50 °C for 2 min, initial denaturation at 95 °C for 2 min, followed by 40 cycles of denaturation at 95 °C for 15 s and annealing/extension at 60 °C for 1 min. Melt curve analysis was performed immediately after amplification (95 °C for 15 s, 60 °C for 1 min, followed by a ramp to 95 °C at 0.3 °C/s). No-template controls (NTCs) containing water instead of cDNA were included in each run to monitor contamination. The -RT controls were also run to ensure no significant amplification from potential genomic DNA contamination.

### 2.6. Protein Extraction and Enzyme-Linked Immunosorbent Assay (ELISA)

Following 5 days of treatment with 1 µM ATRA or vehicle (DMSO), neurospheres were collected by centrifugation (300× *g*, 5 min), washed once with ice-cold PBS, and cell pellets were lysed in RIPA buffer (Thermo Fisher Scientific, Cat# 89900) supplemented with protease inhibitor cocktail (Sigma-Aldrich, Cat# P8340) on ice for 30 min with intermittent vortexing. Lysates were clarified by centrifugation at 14,000× *g* for 15 min at 4 °C. Total protein concentration in the supernatants was determined using the Pierce™ BCA Protein Assay Kit (Thermo Fisher Scientific, Cat# 23225) according to the manufacturer’s instructions.

Protein levels of SOX2, Nestin, and MGMT were quantified using commercially available sandwich ELISA kits: SOX2 (ab245707, Abcam, Cambridge, UK), Nestin (EH334RB, Thermo Fisher Scientific) and MGMT (ab284030, Abcam), following the manufacturers’ protocols. All three kits are validated for human lysates only, show no reported cross-reactivity with rodent proteins, and exhibit intra-/inter-assay CVs < 11%. Limits of detection (LODs) are 31 pg mL^−1^ (SOX2), 0.32 ng mL^−1^ (Nestin), and 4.5 ng mL^−1^ (MGMT). Approximately 50–100 µg of total protein per sample, diluted as per kit instructions, was assayed in duplicate according to the respective manufacturer’s protocols. Absorbance was read at 450 nm on a microplate reader. Standard curves were generated using recombinant protein standards provided with each kit. Protein concentrations were interpolated from the standard curve and normalized to the total protein input, expressed as pg of target protein per µg of total protein lysate (pg/µg). Representative standard curves for the SOX2, Nestin, and MGMT ELISAs are provided in Supplementary Figure S1. Experiments were performed using the same three biological replicates prepared for RNA isolation. ELISA outcomes (pg protein µg^−1^ total protein) were analyzed identically to the mRNA data by unpaired two-tailed Student’s *t*-tests (n = 3).

### 2.7. Data Analysis

Raw amplification data (Ct values) were exported from the StepOnePlus™ Software v2.3. Data processing and analysis were performed using Microsoft Excel and GraphPad Prism v10.4.0. The stability of housekeeping genes (GAPDH, ACTB) across treatment conditions was verified by confirming low variance in their Ct values across all samples. The geometric mean of the Ct values for GAPDH and ACTB was calculated for each sample and used for normalization.

Relative gene expression was calculated using the comparative Ct (ΔΔCt) method [15].

1. Normalization: ΔCt = Ct_Target − Ct_Housekeeping_Mean.
2. Calibration: ΔΔCt = ΔCt_Sample − ΔCt_Vehicle_Average.
3. Relative Quantification (Fold Change): Fold Change = 2^−ΔΔCt. The average fold change for the vehicle control group was set to 1.

### 2.8. Statistical Analysis

Statistical analysis was performed on the ΔCt values using unpaired, two-tailed Student’s *t*-tests to compare the ATRA-treated group versus the vehicle control group for each gene within each cell line. Similarly, protein concentrations determined by ELISA were compared between ATRA-treated and vehicle control groups using unpaired, two-tailed Student’s *t*-tests. Biological replicates (n = three per condition per cell line) were used for statistical comparisons. Results are presented as mean fold change ± Standard Error of the Mean (SEM). Student’s *t*-tests were deemed appropriate for comparing two groups (ATRA-treated vs. vehicle control) for each gene within each cell line in this study. It is recognized that for future studies involving more than two experimental groups or multiple factors, more complex statistical analyses, such as ANOVA with appropriate post hoc tests, would be employed to ensure robustness and account for multiple comparisons.

## 3. Results

### 3.1. RNA Quality and qPCR Validation

Total RNA isolated from U87-MG and A172 cells treated with either vehicle (DMSO) or 1 µM ATRA for 5 days was of high quality, with A260/280 ratios consistently between 1.95 and 2.05 and A260/230 ratios above 1.9. qPCR analysis demonstrated reliable amplification, with single peaks observed in melt curve analyses for all primer sets, confirming amplification specificity. No amplification was detected in NTC wells. Amplification in -RT controls was negligible (Ct > 35 or undetectable), confirming minimal genomic DNA contamination. The housekeeping genes GAPDH and ACTB exhibited stable expression across all experimental conditions, validating their use for normalization.

### 3.2. ATRA Treatment Downregulates Stemness Marker Expression and Protein Levels in GBM Cell Lines

**Figure 1:**
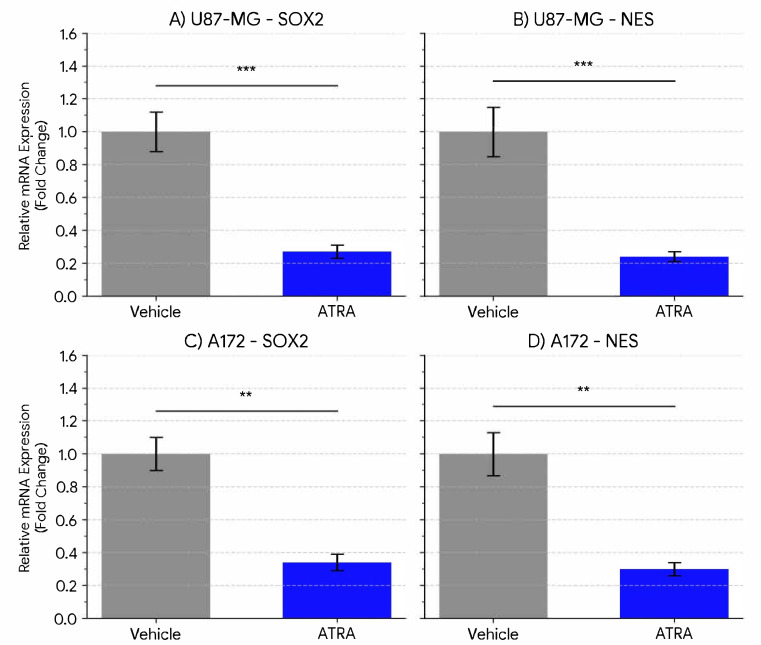
ATRA treatment reduces the expression of stemness markers SOX2 and NES in GBM cell lines. Relative mRNA expression levels of (**A**) SOX2 and (**B**) NES in U87-MG cells, and (**C**) SOX2 and (**D**) NES in A172 cells, following treatment with vehicle (DMSO) or 1 µM ATRA for 5 days. Expression was quantified by qPCR, normalized to the geometric mean of GAPDH and ACTB, and calculated relative to the vehicle control group (set to 1) using the ΔΔCt method. Data are presented as mean fold change ± SEM from three biological replicates. Statistical significance was determined by Student’s *t*-test. ** *p* < 0.01, *** *p* < 0.001.

**Figure 2:**
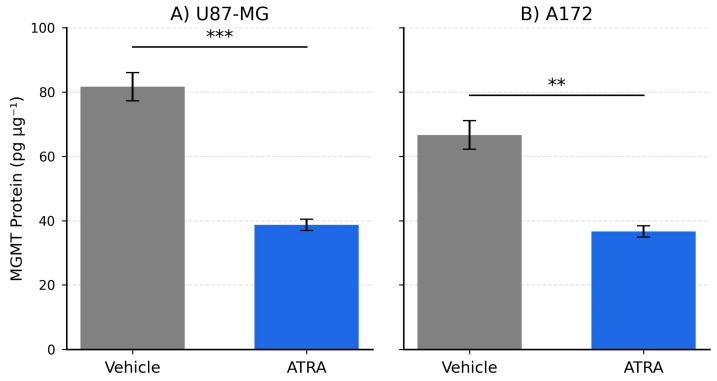
U87-MG and A172 protein validation. Consistent with the mRNA findings, ELISA quantification demonstrated robust reductions at the protein level (Figure 2). In U87-MG, SOX2 protein declined from 148.33 ± 6.01 to 49.67 ± 2.73 pg µg^−1^ (0.335-fold, *p* = 0.0002) and Nestin from 450.00 ± 17.32 to 128.33 ± 4.41 pg µg^−1^ (0.285-fold, *p* = 0.00008). A172 showed analogous decreases—SOX2: 123.33 ± 5.87 to 53.33 ± 2.40 pg µg^−1^ (0.432-fold, *p* = 0.0007); Nestin: 403.33 ± 14.53 to 144.33 ± 3.53 pg µg^−1^ (0.358-fold, *p* = 0.00009). ** *p* < 0.01, *** *p* < 0.001.

**Figure 3:**
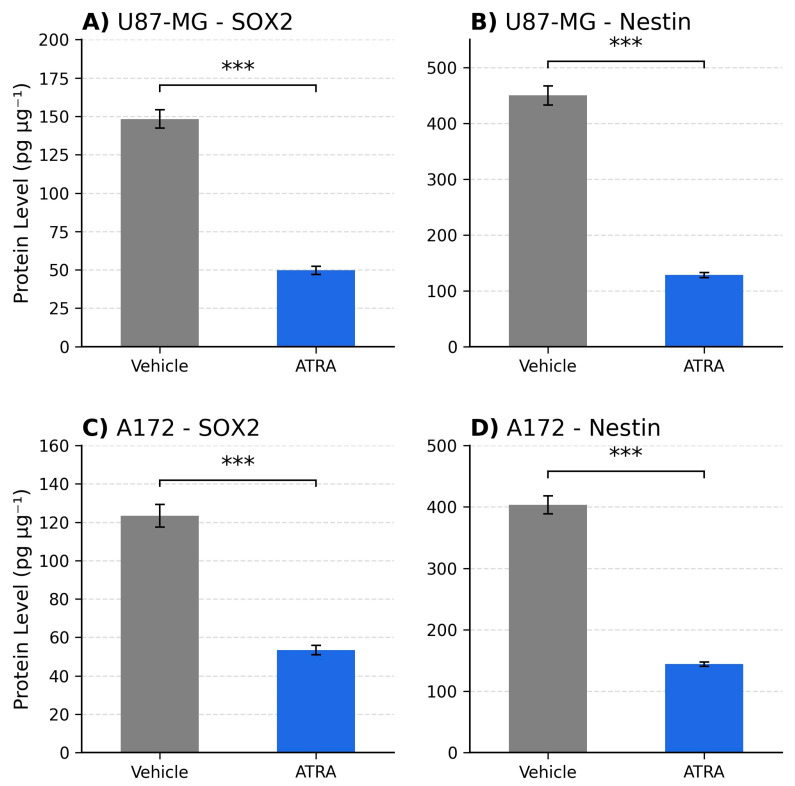
MGMT protein. ATRA also reduced MGMT protein (Figure 3). U87-MG levels fell from 81.67 ± 4.41 to 38.67 ± 1.76 pg µg^−1^ (0.473-fold, *p* = 0.0005) and A172 from 66.67 ± 4.41 to 36.67 ± 1.76 pg µg^−1^ (0.550-fold, *p* = 0.0015). *** *p* < 0.001.

To determine the effect of ATRA on stemness-associated markers in GBM cells cultured under neurosphere conditions, we quantified the mRNA expression of the core stemness transcription factor SOX2 and the intermediate filament protein Nestin (NES). In the U87-MG cell line, treatment with 1 µM ATRA for 5 days resulted in a highly significant decrease in the expression of both markers compared to vehicle-treated controls. Figure 1A shows that SOX2 mRNA levels were reduced by an average of 3.7-fold (Mean ± SEM: Vehicle 1.00 ± 0.12 vs. ATRA 0.27 ± 0.04; *p* = 0.0008). Similarly, as depicted in Figure 1B, NES mRNA expression was significantly downregulated by 4.1-fold following ATRA treatment (Vehicle 1.00 ± 0.15 vs. ATRA 0.24 ± 0.03; *p* = 0.0005). Similar effects were observed in the A172 cell line. Figure 1C illustrates that ATRA treatment led to a significant reduction in SOX2 mRNA expression by 2.9-fold (Vehicle 1.00 ± 0.10 vs. ATRA 0.34 ± 0.05; *p* = 0.0041), and Figure 1D demonstrates a significant decrease in NES mRNA expression by 3.3-fold (Vehicle 1.00 ± 0.13 vs. ATRA 0.30 ± 0.04; *p* = 0.0028). These results demonstrate that ATRA treatment effectively suppresses the expression of key genes associated with stem-like states in both established GBM lines when cultured under these conditions. As shown in Figure 2, SOX2 protein declined from 148.3 ± 6.0 to 49.7 ± 2.7 pg µg^−1^ (*p* = 0.0002) and Nestin from 450.0 ± 17.3 to 128.3 ± 4.4 pg µg^−1^ (*p* = 0.00008). ATRA also reduced MGMT protein levels in both lines. In U87-MG, MGMT fell from 81.67 ± 4.41 to 38.67 ± 1.76 pg µg^−1^ (0.473-fold; *p* = 0.0005), and in A172 from 66.67 ± 4.41 to 36.67 ± 1.76 pg µg^−1^ (0.550-fold; *p* = 0.0015) (Figure 3).

### 3.3. ATRA Treatment Downregulates MGMT Expression and Protein Levels in GBM Cell Lines

Given the critical role of MGMT in mediating TMZ resistance, we investigated whether ATRA treatment also modulates MGMT expression in these cell line models. We quantified MGMT mRNA levels in U87-MG and A172 cells following 5 days of vehicle or ATRA treatment.

**Figure 4:**
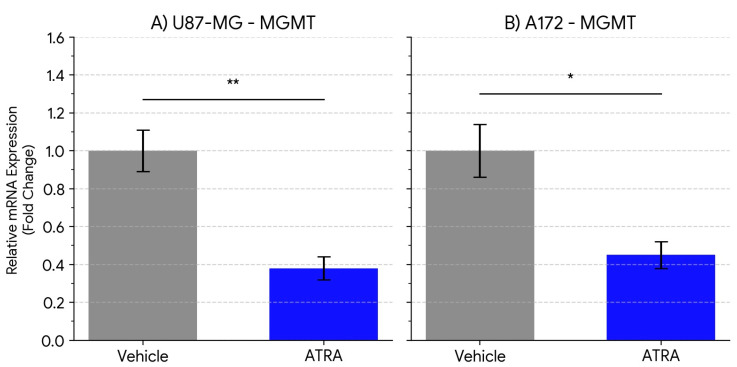
ATRA treatment reduces the expression of the TMZ resistance gene MGMT in GBM cell lines. Relative mRNA expression levels of MGMT in (**A**) U87-MG cells and (**B**) A172 cells following treatment with vehicle (DMSO) or 1 µM ATRA for 5 days. Expression was quantified by qPCR, normalized to the geometric mean of GAPDH and ACTB, and calculated relative to the vehicle control group (set to 1) using the ΔΔCt method. Data are presented as mean fold change ± SEM from three biological replicates. Statistical significance was determined by Student’s *t*-test. * *p* < 0.05, ** *p* < 0.01.

In U87-MG cells, ATRA treatment resulted in a statistically significant decrease in MGMT mRNA expression compared to the vehicle control group. As shown in Figure 4A, the average MGMT transcript level was reduced by 2.6-fold (Vehicle 1.00 ± 0.11 vs. ATRA 0.38 ± 0.06; *p* = 0.0065).

A similar significant downregulation of MGMT expression was observed in the A172 cell line following ATRA treatment. Figure 4B depicts that MGMT mRNA levels decreased by an average of 2.2-fold compared to vehicle controls (Vehicle 1.00 ± 0.14 vs. ATRA 0.45 ± 0.07; *p* = 0.0132).

These findings indicate that ATRA treatment, concurrently with reducing stemness marker expression, also reduces the expression of the key TMZ resistance gene MGMT at the transcript level in both tested GBM cell lines under neurosphere culture conditions.

### 3.4. Summary of qPCR Results

The relative fold changes and statistical significance for all target genes in both cell lines are summarized in Table 2.

MGMT protein declined from 81.67 ± 4.41 to 38.67 ± 1.76 pg µg^−1^ (0.473-fold; *p* = 0.0005), and in A172 from 66.67 ± 4.41 to 36.67 ± 1.76 pg µg^−1^ (0.550-fold; *p* = 0.0015) (Table 3).

## 4. Discussion

The resistance of glioblastoma to conventional therapies, particularly TMZ, represents a major obstacle in treating this disease. This study investigated the potential of ATRA-induced differentiation to concomitantly reduce stemness-associated properties and modulate the expression of the critical TMZ resistance gene, MGMT, in established human GBM cell lines cultured under neurosphere-promoting conditions. Our findings demonstrate, at both the mRNA and protein level, that treatment with 1 µM ATRA for 5 days is associated with a significant downregulation of the expression of key stemness markers SOX2 and NES, as well as MGMT, in both U87-MG and A172 cell lines under these specific in vitro conditions.

The observed downregulation of SOX2 and NES (approx. 3- to 4-fold reduction) suggests that ATRA treatment promotes a shift away from a stem-like transcriptional state in these models. Our ELISA data confirm that these transcriptional changes translate into marked protein depletion, with ~3- to 3.5-fold reductions for SOX2 and Nestin in U87-MG and ~2- to 2.8-fold in A172. This concordance strengthens the biological relevance of ATRA-driven differentiation cues beyond gene expression. SOX2 is a master regulator crucial for maintaining stem cell identity, including in glioma [7,8]. Nestin is associated with neural progenitors and a more aggressive phenotype in GBM [9,24]. The reduction in transcripts for both factors aligns with previous reports indicating that ATRA can induce morphological and molecular changes consistent with differentiation in glioma cell lines [2,20,25]. Our quantitative data at the transcript level support these observations in established lines cultured under conditions designed to enrich stem-like features, suggesting ATRA has the potential to modulate these specific pathways in these models.

Our findings of ATRA-induced downregulation of SOX2 and Nestin mRNA are broadly consistent with previous reports indicating ATRA can induce differentiation in various glioma cell line models [2,20,25]. While some studies have suggested ATRA can sensitize glioma cells to chemotherapy or radiation [22,23], the specific concurrent downregulation of MGMT mRNA alongside key stemness markers in GBM cells cultured under neurosphere conditions, as observed here, adds to the understanding of ATRA’s potential multifaceted impact. In other cancer types, such as acute promyelocytic leukemia, ATRA’s success is well-established [18], and studies in neuroblastoma have also shown that retinoids can induce differentiation and modulate therapy resistance pathways. The current study provides specific molecular data in GBM models suggesting a dual effect on stemness-associated transcripts and a key chemoresistance gene, MGMT, warranting further investigation into how these transcriptional changes translate to functional sensitization.

Perhaps the most significant finding of this study is the concurrent downregulation of MGMT mRNA expression following ATRA treatment (approx. 2.2- to 2.6-fold reduction). MGMT is the primary determinant of TMZ resistance in GBM, and its expression level is inversely correlated with treatment response [12,13,14]. While differentiation therapy has been proposed as a means to sensitize cancer cells, direct evidence linking ATRA to the modulation of MGMT expression in GBM models has been limited. Our results show a clear reduction at the mRNA level of MGMT transcripts associated with ATRA treatment in U87-MG and A172 cells. The 2.1- to 1.8-fold reduction we observed at the MGMT protein level mirrors the transcript data, implying that ATRA can diminish actual repair capacity, not merely transcriptional potential. This finding provides a potential molecular mechanism by which ATRA could modulate pathways associated with TMZ sensitivity, particularly relevant for tumors expressing significant levels of MGMT. This could be important as many GBM patients present with unmethylated MGMT promoters and derive limited benefit from TMZ [15].

The precise molecular mechanisms by which ATRA downregulates MGMT mRNA expression remain yet to be fully elucidated from our data. While we speculate that ATRA signaling via RAR/RXR heterodimers might directly or indirectly repress MGMT transcription [19,26,27], or that ATRA-induced differentiation leads to broader epigenetic reprogramming, these are hypotheses requiring direct experimental validation. Future studies should aim to investigate these mechanisms, for example, by using chromatin immunoprecipitation (ChIP) assays to assess potential direct binding of RAR to regulatory elements within the MGMT promoter or enhancer regions. Additionally, analyzing changes in histone modifications at the MGMT locus following ATRA treatment would provide further mechanistic insights

Beyond direct promoter repression, ATRA may converge on several signaling axes that co-regulate stemness and MGMT. RAR/RXR activation can attenuate STAT3 and c-Myc activity, both of which sustain SOX2 expression. Retinoids also oppose Notch and Wnt/β-catenin pathways that maintain glioma stem-cell self-renewal, while simultaneously up-regulating BMP4, a driver of astro-glial differentiation. Crosstalk among these networks provides a plausible route whereby differentiation cues lower MGMT transcription indirectly through chromatin relaxation and decreased Sp1 occupancy at the MGMT enhancer.

We observed the consistent effects of ATRA on SOX2, NES, and MGMT expression across two distinct established GBM lines, U87-MG and A172, strengthening the potential generalizability of these findings within these specific model systems. Although the magnitude of downregulation varied slightly, the overall trend was robustly significant in both.

It is crucial, however, to acknowledge the limitations of this study. Firstly, we used two established cell lines (U87-MG, A172) cultured under specific neurosphere conditions. While useful and widely employed, these models may not fully recapitulate the biological heterogeneity and complex characteristics of patient-derived GSCs or primary GBM tumors in situ. Future studies should aim to validate these findings using patient-derived glioblastoma stem-like cell (GSC) lines, which may better reflect the heterogeneity and biology of primary tumors and enhance the translational relevance of these observations. Also, these experiments were conducted in vitro. The tumor microenvironment in vivo could significantly influence cellular responses to ATRA. Furthermore, the statistical power of the current study is based on a sample size of n = three biological replicates per condition. While Student’s *t*-tests were appropriate for the direct two-group comparisons made, future investigations with larger sample sizes would provide greater statistical robustness. Finally, investigating a broader range of ATRA concentrations, time points, and additional differentiation/stemness markers would provide a more comprehensive understanding. Future studies should also aim to validate these findings in orthotopic xenograft models, assessing tumor growth, marker expression, and the combinatorial efficacy of ATRA and TMZ in vivo.

Despite these limitations, our findings have significant implications. They provide a strong molecular rationale supporting the further exploration of ATRA as a modulator of key pathways in GBM models. By simultaneously reducing the mRNA expression of stemness-associated markers and the MGMT gene in these established GBM cell lines, ATRA demonstrates the potential at the transcriptional level for impacting both stem-like properties and TMZ resistance mechanisms. This warrants further preclinical investigation into ATRA, potentially in combination with TMZ, assessing functional outcomes such as effects on cell proliferation, clonogenicity, TMZ sensitivity (IC50 determination), and ultimately, in vivo efficacy in appropriate GBM models.

## 5. Conclusions

In summary, this study demonstrated that All-Trans Retinoic Acid (ATRA) treatment significantly reduces the expression of the key stemness markers SOX2 and Nestin in the established glioblastoma cell lines U87-MG and A172, when cultured under neurosphere-promoting conditions. Critically, our data show that ATRA concurrently decreases the mRNA expression of the pivotal DNA repair and temozolomide resistance gene, MGMT, in these models. These transcriptional findings in vitro provide a molecular rationale for further investigating ATRA’s potential. Key next steps should include: (1) quantify MGMT enzymatic activity and determine whether protein down-regulation translates into functional sensitization to temozolomide (TMZ) in vitro; (2) investigating the in vivo efficacy of ATRA, both alone and in combination with TMZ, using relevant orthotopic GBM models; and (3) extending these investigations to patient-derived GSC models to better assess translational potential. Such studies are warranted to determine if these initial molecular observations can translate into effective therapeutic strategies for glioblastoma. The concordant protein data presented here satisfy a key prerequisite for progression to functional MGMT activity assays and TMZ-sensitization studies.

## Figures and Tables

**Table 1:** Primer Sequences for qPCR.

Gene Symbol	Gene Name	Forward Primer (5′-3′)	Reverse Primer (5′-3′)	Amplicon Size (bp)
SOX2	SRY-Box Transcription Factor 2	GCTACAGCATGATGCAGGACCA	TCTGCGAGCTGGTCATGGAGTT	145
NES	Nestin	GGCGCACCTCAAGATGTCCCT	CAGGGAAGAGGTGGGAGACAAG	128
MGMT	O-6-Methylguanine-DNA Methyltransferase	GGTCTGCGAAGAGGAGGAAGG	CACCCAGTCGGAGGATAAGTTG	110
GAPDH	Glyceraldehyde-3-Phosphate Dehydrogenase	GTCTCCTCTGACTTCAACAGCG	ACCACCCTGTTGCTGTAGCCAA	122
ACTB	Beta-Actin	CACCATTGGCAATGAGCGGTTC	AGGTCTTTGCGGATGTCCACGT	165

**Table 2:** Summary of Relative Gene Expression Changes Following 1 µM ATRA Treatment (5 days).

Cell Line	Target Gene	Mean Fold Change (ATRA vs. Vehicle) ± SEM	*p*-Value (vs. Vehicle)
U87-MG	SOX2	0.27 ± 0.04	0.0008
U87-MG	NES	0.24 ± 0.03	0.0005
U87-MG	MGMT	0.38 ± 0.06	0.0065
A172	SOX2	0.34 ± 0.05	0.0041
A172	NES	0.30 ± 0.04	0.0028
A172	MGMT	0.45 ± 0.07	0.0132

Data represent mean ± SEM from n = three biological replicates. Fold change is relative to the vehicle control group (set to 1.0). *p*-values were calculated using Student’s *t*-test comparing ΔCt values between ATRA and vehicle groups.

**Table 3:** Summary of Relative Protein Level Changes Following 1 µM ATRA Treatment (5 days).

Cell Line	Protein	Vehicle (pg µg^−1^ ± SEM)	ATRA (pg µg^−1^ ± SEM)	Fold Change (ATRA/Vehicle)	*p*-Value	Significance
U87-MG	SOX2	148.33 ± 6.01	49.67 ± 2.73	0.335 (2.99-fold ↓)	0.0002	***
U87-MG	Nestin	450.00 ± 17.32	128.33 ± 4.41	0.285 (3.51-fold ↓)	0.00008	***
U87-MG	MGMT	81.67 ± 4.41	38.67 ± 1.76	0.473 (2.11-fold ↓)	0.0005	***
A172	SOX2	123.33 ± 5.87	53.33 ± 2.40	0.432 (2.31-fold ↓)	0.0007	***
A172	Nestin	403.33 ± 14.53	144.33 ± 3.53	0.358 (2.79-fold ↓)	0.00009	***
A172	MGMT	66.67 ± 4.41	36.67 ± 1.76	0.550 (1.82-fold ↓)	0.0015	**

*** *p* < 0.001 and ** *p* < 0.01 and down arrow is decrease.

## Data Availability

Due to institutional data management requirements, further datasets are available from the corresponding author upon reasonable request.

## Supplementary Materials

The following supporting information can be downloaded at: https://www.mdpi.com/article/10.3390/cells14100746/s1, Figure S1: Representative standard curves for SOX2, Nestin, and MGMT ELISAs.
