# Supplementary material for: All-Trans Retinoic Acid Induces Differentiation and Downregulates Stemness Markers and MGMT Expression in Glioblastoma Stem Cells
Source: Cells. 2025 May 20;14(10):746. doi: 10.3390/cells14100746 (PMC12109622; doi:10.3390/cells14100746)
Supplement: Supplementary file 1 [file cells-14-00746-s001.zip › cells-3612945-supplementary.pdf]

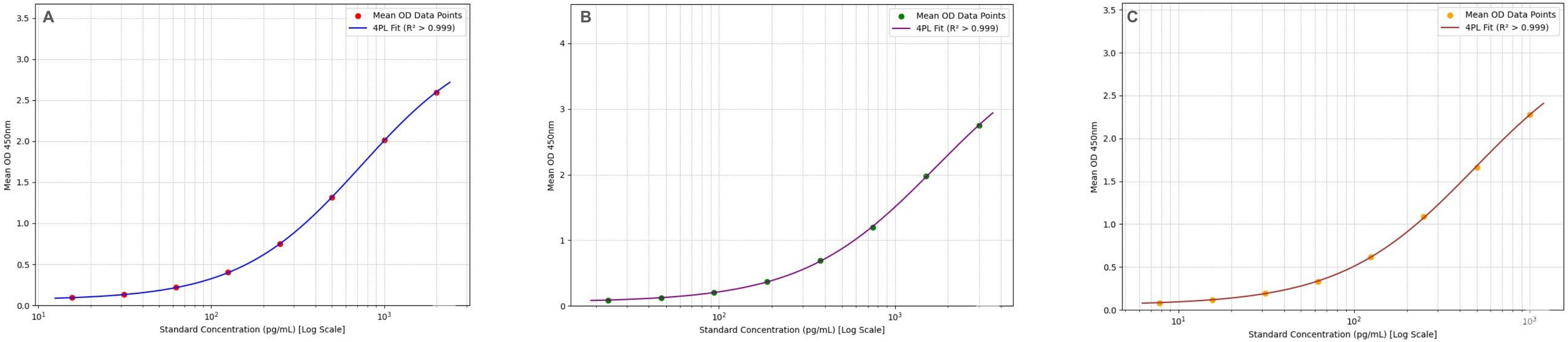


**Supplementary Figure S1. Representative standard curves for SOX2, Nestin, and MGMT ELISAs:** Standard curves used for the quantification of (A) SOX2, (B) Nestin, and (C) MGMT protein levels by Enzyme-Linked Immunosorbent Assay (ELISA). Each curve plots the mean optical density (OD) at 450 nm against known concentrations of the respective recombinant protein standard (pg/mL) on a logarithmic x-axis. Data points were fitted using a four-parameter logistic (4PL) regression model. All fits showed a high coefficient of determination ($R^2 > 0.999$), indicating a strong fit of the model to the data.
